# Targeting Human Endothelial Cells with Glutathione and Alanine Increases the Crossing of a Polypeptide Nanocarrier through a Blood–Brain Barrier Model and Entry to Human Brain Organoids

**DOI:** 10.3390/cells12030503

**Published:** 2023-02-03

**Authors:** Mária Mészáros, Thi Ha My Phan, Judit P. Vigh, Gergő Porkoláb, Anna Kocsis, Emese K. Páli, Tamás F. Polgár, Fruzsina R. Walter, Silvia Bolognin, Jens C. Schwamborn, Jeng-Shiung Jan, Mária A. Deli, Szilvia Veszelka

**Affiliations:** 1 Institute of Biophysics, Biological Research Centre, Eötvös Loránd Research Network, H-6726 Szeged, Hungary; 2 Department of Chemical Engineering, National Cheng Kung University, Tainan 70101, Taiwan; 3 Doctoral School of Biology, University of Szeged, H-6720 Szeged, Hungary; 4 Theoretical Medicine Doctoral School, University of Szeged, H-6722 Szeged, Hungary; 5 Luxembourg Centre for Systems Biomedicine (LCSB), Developmental and Cellular Biology, University of Luxembourg, 4365 Belvaux, Luxembourg

**Keywords:** brain endothelial cells, blood–brain barrier, brain organoid, peptide nanocarriers, 3-armed polypeptides, alanine, glutathione, dual-targeting

## Abstract

Nanoparticles (NPs) are the focus of research efforts that aim to develop successful drug delivery systems for the brain. Polypeptide nanocarriers are versatile platforms and combine high functionality with good biocompatibility and biodegradability. The key to the efficient brain delivery of NPs is the specific targeting of cerebral endothelial cells that form the blood–brain barrier (BBB). We have previously discovered that the combination of two different ligands of BBB nutrient transporters, alanine and glutathione, increases the permeability of vesicular NPs across the BBB. Our aim here was to investigate whether the combination of these molecules can also promote the efficient transfer of 3-armed poly(l-glutamic acid) NPs across a human endothelial cell and brain pericyte BBB co-culture model. Alanine and glutathione dual-targeted polypeptide NPs showed good cytocompatibility and elevated cellular uptake in a time-dependent and active manner. Targeted NPs had a higher permeability across the BBB model and could subsequently enter midbrain-like organoids derived from healthy and Parkinson’s disease patient-specific stem cells. These results indicate that poly(l-glutamic acid) NPs can be used as nanocarriers for nervous system application and that the right combination of molecules that target cerebral endothelial cells, in this case alanine and glutathione, can facilitate drug delivery to the brain.

## 1. Introduction

The design of carrier systems and the proper nanoformulations of drug molecules are key problems to be solved to treat disorders of the central nervous system (CNS) [1]. The blood–brain barrier (BBB), present at the level of brain capillaries and microvessels, limits the penetration of drugs, particularly biomolecules, from the blood circulation into the brain tissue [2]. Despite the initial success in the research of promising biological drugs, several clinical trials failed because of the low penetration of the neurotherapeutic candidates across the BBB [3]. Nanosized synthetic or natural drug delivery systems, such as lipid, metal, carbon and polymer-based nanoparticles (NPs), are extensively investigated to enhance drug delivery across the BBB [1,4,5]. The major requirements for a therapeutic nanoformulation, low toxicity, high biodegradability, high drug-loading capacity, low immunogenicity and long half-time in the circulation, are not met by most nanoconstructs developed for CNS application [6].

Natural and synthetically prepared polypeptides or polyamino acids are versatile candidates for the development of brain-specific nanocarriers [7]. Polypeptides combine extensive functionality with biocompatibility and biodegradability [8,9]. The ideal structure of polypeptide nanoconjugates consists of a biodegradable polymer matrix, an active agent (drugs), a linker, and targeting molecule(s) [8]. The high drug coupling capacity of polypeptides also provides a wide applicability, especially for brain targeting [7,10]. Analogs of poly(glutamate)s are broadly used in the food and cosmetic industry, due to their favorable physico-chemical properties, such as hydrophilicity, hydrogel formation and their capacity for electrostatic interactions. The most known isoform of poly(glutamate) is the natural poly(γ-glutamic acid), present in high amounts in the Japanese health food, natto, which is produced by *Bacillus subtilis* [11]. Another isoform is the synthetic poly(l-glutamic acid) (PLG), produced by ring-opening polymerization by N-carboxyanhydrides [11,12]. The reactive terminal carboxylic function groups of PLGs allow the covalent coupling of drugs to the molecules; therefore, their conjugates are suitable for biomedical applications [11]. PLGs have already been used in drug delivery systems, as theranostic or antiviral agents [7,12,13], but these polymers were scarcely investigated for brain applications.

Despite intensive research efforts to develop nanocarrier systems for brain delivery, there are still no NP-based therapeutics for CNS diseases. Drug loading into NPs alone is not enough for the successful delivery of drugs to the CNS [14]. To elevate the permeability of nanocarriers across the BBB, specific targeting is needed [15,16]. Shuttle molecules, such as cell-penetrating peptides, can elevate the penetration of NPs across biological barriers via receptor-mediated or adsorptive-mediated transcytosis [17], but with poor brain specificity. Influx transport systems are highly expressed on the cerebral endothelium [18,19,20,21], and some of them have been exploited by using their respective ligands to target the CNS. They play a physiological role in nutrient delivery via receptor-mediated endocytosis, adsorptive-mediated endocytosis and carrier-mediated transport by solute carriers [2,22]. In preclinical studies, the receptor-mediated transport systems were mostly investigated by decorating the surface of NPs with the ligands of BBB receptors [15,23]; these include various types of apolipoproteins, the ligands of the low-density lipoprotein receptor or low-density lipoprotein-associated receptors [24,25,26], transferrin, its peptides or anti-transferrin antibodies [27,28], and insulin or anti-insulin antibodies [29]. High affinity influx transport to the brain was described for the tripeptide glutathione (GSH) [30], which led to the development of a brain-targeting platform [31]. Clinical studies were conducted on GSH-targeted liposomes loaded with doxorubicin [32] and methylprednisolone [33]. GSH, as a successful brain-targeting ligand, is used in many preclinical studies; our group also demonstrated that GSH enhances the delivery of NPs across a BBB model [34] via specific docking to the brain endothelial surface [35].

In the research field of nanocarriers, the functionalization of NPs with the ligands of nutrient transporters is still underrepresented compared to the targeting of BBB receptors. The expression level of hexose transporters is high on brain endothelial cells and they can be targeted by glucose or glucose analogs. Indeed, glucose or glucose analog labeling elevates the BBB crossing of vesicular [36,37,38] or metal [39,40,41] NPs in cell culture and animal studies. Besides glucose, l-DOPA, targeting the amino acid carrier LAT1/SLC7A5 [42], and biotin, the ligand of a multivitamin transporter (SMVT/SLC5A6) of the BBB, can also be used as NP-targeting molecules. Biotin labeling increases both the cellular uptake of solid fluorescent polystyrene NPs and their transfer across a human culture model of the BBB [34]. Since the expression pattern of transporters on the luminal membrane of brain endothelial cells is BBB specific, our group hypothesizes that combinations of ligands of nutrient transporters can elevate the brain specificity and delivery of NPs. Our group targeted vesicular NPs with the dual combinations of GSH, glucopyranose and alanine (A), and found that a combination of two different molecules, especially A and GSH, resulted in a better uptake into brain endothelial cells and a more efficient penetration across the BBB [43,44]. The combined A–GSH targeting of vesicular NPs also leads to a higher uptake into brain pericytes, astrocytes and neurons [44].

The aim of the present proof-of-concept study was to investigate whether the A–GSH targeting molecule combination that enhances the BBB penetration of vesicular NPs can be used for a polypeptide nanocarrier, in order to increase cellular uptake and BBB permeability. Therefore, 3-armed poly(l-glutamic acid) nanocarriers were functionalized with A and GSH molecules in order to investigate internalization in human brain endothelial cells, the uptake mechanism, and permeability across a human culture model of BBB. Finally, we explored whether the dual-targeted polypeptide NPs, after crossing the human BBB model, can penetrate into midbrain organoids, differentiated from healthy and Parkinson’s disease patient-specific stem cells.

## 2. Materials and Methods

### 2.1. Materials

All the chemicals used were purchased from Merck Life Science Kft., Budapest, Hungary, unless otherwise indicated.

### 2.2. Synthesis and Characterization of Polypeptide Nanocarriers

**Figure 1:**
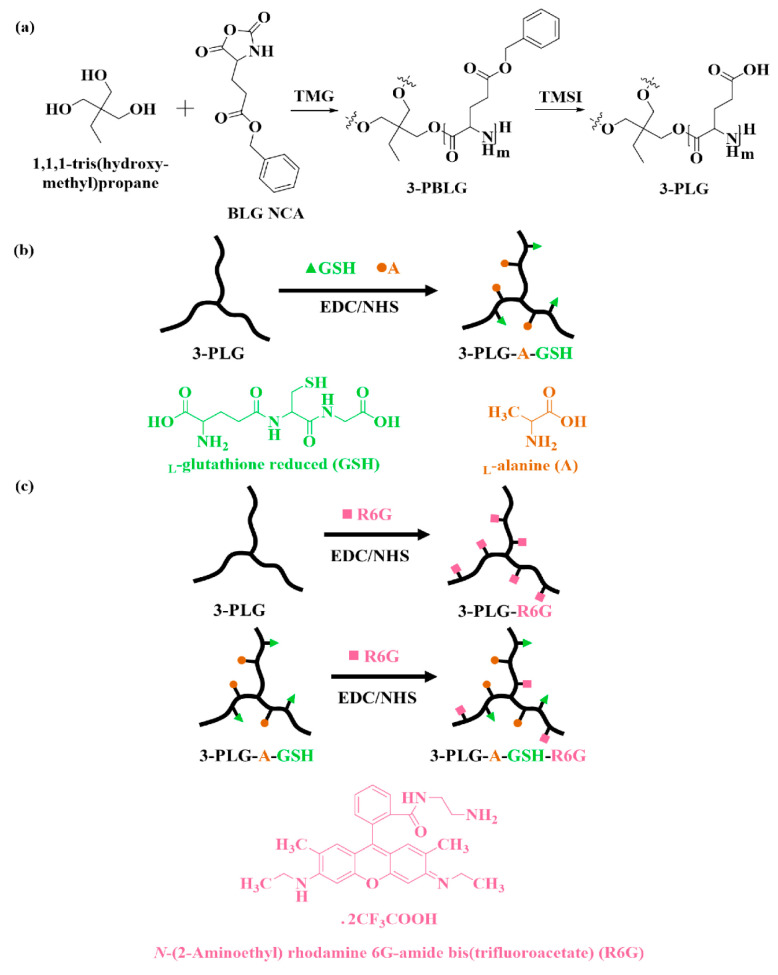
(**a**) Synthesis of 3-armed poly(l-glutamic acid γ-benzyl ester) (3-PBLG) and deprotection of benzyl groups to prepare 3-armed poly(l-glutamic acid) (3-PLG), (**b**) Synthesis of l-alanine (A) and glutathione (GSH)-targeted, 3-armed poly(l-glutamic acid) (3-PLG-A–GSH), (**c**) Synthesis of rhodamine 6G (R6G), labeled 3-PLG-R6G or 3-PLG-A–GSH-R6G.

The 3-armed poly(γ-benzyl-l-glutamic acid) (3-PBLG) was synthesized by using the procedure reported in our previous paper [45]. In this study, 1,1,1-tris(hydroxymethyl)propane and 1,1,3,3-tetramethylguanidine (TMG) were used as the 3-armed initiator and the promoter for ring-opening polymerization, respectively (Figure 1a) [46,47].

3-PBLG with degree of polymerization (20) was synthesized by the following procedure: the 3-armed initiator (17.0 mg) and l-glutamic acid γ-benzyl ester N-carboxyanhydride (BLG NCA; 2.0 g) were separately dissolved in 6.3 mL and 7.6 mL of anhydrous dimethylformamide (DMF). TMG (28.5 μL) was added into the initiator solution, heated and stirred for 30 min. The BLG NCA solution was added into the initiator solution before taking it out of the glovebox. Upon stirring at 30 °C for 3 days, the reaction solution was dialyzed against methanol for 1 day and deionized water (DIW) for 3 days with a dialysis membrane tube (MWCO 6000–8000 Da), before the freeze-drying process. The final product was obtained as a white solid (yield of 90%). The sample was dissolved in trifluoroacetic acid (TFA-*d*_1_) and DMF for ^1^H NMR and GPC-LS analyses, respectively. A Viscotek GPC-LS, using DMF with 0.1 M LiBr as the eluent, was employed to analyze the sample with 1.0 mL/min of the flow rate at 55 °C. ^1^H NMR analysis was carried on an AVANCE III HD 600 NMR.

Iodotrimethylsilane (TMSI) was used to remove the benzyl group on the BLG segment for the deprotection of PBLG (Figure 1a) [45]. The polypeptide (1.0 g) was dissolved in 100 mL of anhydrous dichloromethane completely before TMSI was added into the solution, in the dark, with 5-fold mole of benzyl group. The reactor was covered by aluminum foil and stirred at 40 °C for 24 h before the precipitation in 900 mL of hexane. The obtained solid was dissolved in basic water, dialyzed against DIW for 3 days (MWCO 3500 Da) and freeze dried for 3 days. The lyophilized sample was dissolved in deuterium oxide (D_2_O) for ^1^H NMR analysis.

To modify the nanocarriers with different functional groups, 3-armed poly(l-glutamic acid) (3-PLG) was grafted with l-alanine (A) and l-glutathione (GSH) by using the N-ethyl-N′-(3-(dimethylamino)propyl)carbodiimide/N-hydroxysuccinimide (EDC/NHS) coupling reaction (Figure 1b) [12,48]. The molar ratio of l-glutamic acid to A and GSH was set as 1:0.5:0.5. After each step, the polymer was dialyzed against DIW before lyophilization. The freeze-dried 3-PLG and A–GSH dual-targeted 3-PLG (3-PLG-AGSH) samples were dissolved in D_2_O for ^1^H NMR analysis. Then, 3-PLG and 3-PLG-A-GSH were grafted with N-(2-Aminoethyl) rhodamine 6G-amide bis(trifluoroacetate) (R6G) by using the EDC/NHS coupling reaction (Figure 1c). The weight ratio of 3-PLG to R6G was set as 1:20.

The morphology of the 3-PLG and 3-PLG-A-GSH samples was evaluated by transmission electron microscopy. Samples (20 μL, 6 mg/mL) were dropped on formvar-coated 150-mesh copper grids, and excessive fluid was removed with the edge of a filter paper after 1 min. Samples were contrasted with 10 μL of uranyl acetate (1%, Electron Microscopy Sciences, Hatfield, PA, USA) in 50% ethanol for 3 min (2 times) and were dried under a Petri dish overnight before the electron microscopic evaluation. The negatively stained nanocarriers were visualized with a JEM-1400 Flash microscope (JEOL Ltd., Tokyo, Japan) at 10,000× magnification. Tag Image File Format (TIFF) images were recorded at 50,000× magnification with the built-in Matataki Flash camera.

The size, polydispersity index (PDI) and zeta potential of the polypeptide nanocarriers were determined by dynamic light scattering measurements (Malvern Zetasizer Nano ZS, equipped with a He–Ne laser (λ = 632.8 nm), Malvern Instruments, UK). Before measurements, samples were diluted in filtered PBS to a final concentration of 0.5 mg/mL. Means were calculated from the average of at least 3 × 13 measurements per sample.

### 2.3. Cell Cultures

Human cord blood stem cell-derived endothelial cells were grown in endothelial cell culture media (ECM-NG, Sciencell, Carlsbad, CA, USA), supplemented with 5% fetal bovine serum (FBS), 1% endothelial growth supplement (ECGS, Sciencell, Carlsbad, CA, USA) and 0.5% gentamicin [49]. Bovine brain pericytes (PCs) were seeded (≤P11) into 0.2% gelatin coated dishes (Corning Costar Co., Corning, MA, USA) in Dulbecco’s modified Eagle’s medium (DMEM, Life Technologies, Carlsbad, CA, USA, Thermo Fisher Scientific, Waltham, MA, USA), supplemented with 20% FBS, 1% Glutamax (Life Technologies, Carlsbad, CA, USA) and gentamicin (0.5%) [49,50]. The conditioned medium was collected from the PC cultures on the second day after the cell seeding. Batches of human endothelial cells and PCs were received from the Laboratoire de la Barrière Hémato-Encéphalique, University of Artois, Lens, France. Co-culture with brain PCs [49] or treatment with PC-conditioned medium [50] induces BBB characteristics in the stem cell-derived human endothelial cells; these are called human brain-like endothelial cells (hBECs).

Midbrain organoids were established from human floor plate neuronal progenitor cells derived from a healthy (ID number: #232) patient and a Parkinson’s disease patient, with triplication in the SNCA gene (ID number: #317) [51]. The generation and maintenance of the organoids are described by Nickels et al. [51].

### 2.4. Measurement of Cellular Viability

#### 2.4.1. Impedance Measurements

The effect of nanocarriers on the viability of hBECs was monitored by the label-free, real-time, non-invasive measurement of cell layer impedance (RTCA-SP instrument, Agilent Technologies, Santa Clara, CA, USA). Changes in the impedance values correlate linearly with cellular viability [44,52]. The hBECs (P6) were seeded at a density of 6 × 10^3^ cells/well in 96-well plates with integrated gold electrodes (E-plate 96, Agilent), coated with 0.2% gelatin. To differentiate hBECs, the cells received a culture medium mixed with a PC-conditioned medium (50–50%) for 48 h. The confluent layers of hBECs in the plateau phase of growth were treated with 3-PLG or 3-PLG-A-GSH nanocarriers (1, 10, 20 or 100 µg/mL), diluted in hBEC culture medium. The impedance was followed every 5 min for 24 h. The cell index was defined as R_n_-R_b_ at each time point of measurement, where R_n_ is the cell-electrode impedance of the well when it contains cells, and R_b_ is the background impedance of the well with the medium alone. The cell index was normalized in each well to the value measured at the last time point before the treatment.

#### 2.4.2. Colorimetric Cytotoxicity Tests

The yellow 3-(4,5-dimethyltiazol-2-yl)-2,5-diphenyltetrazolium bromide (MTT; Sigma M5655) dye is taken up by cells and converted into blue formazan crystals by mitochondrial and cytoplasmic enzymes. Only living cells can convert MTT, so this test determines cell metabolic activity and reflects cell viability [34]. A decrease in dye reduction correlates with cell damage. The hBECs (P6) were seeded at a density of 6 × 10^3^ cells/well in 96-well plates coated with 0.2% gelatin. To differentiate hBECs, the cells received a culture medium mixed with a PC-conditioned medium (50–50%) for 48 h. The confluent layers of hBECs were treated with 3-PLG or 3-PLG-A-GSH nanocarriers (1, 10, 20 or 100 µg/mL), diluted in hBEC culture medium for 24 h. The MTT dye solution was prepared in phenol red-free medium at a 0.5 mg/mL final concentration. After the 1-day incubation with NPs, the medium was removed and the MTT solution was added to the cells. The plates were incubated for 4 h at 37 °C. Formazan crystals, produced by living cells, were dissolved in 100 μL/well dimethyl sulfoxide on a horizontal shaker for 10 min. Dye absorbance was detected by a multi-well plate reader at a 570 nm wavelength (Fluostar Optima, BMG Labtechnologies, Baden-Wuerttemberg, Germany). Cell viability was calculated as the percentage of dye reduction by culture medium-treated cells (control group).

### 2.5. Cellular Uptake of Polypeptide Nanocarriers, Visualization and Mechanisms of Internalization

For the cellular uptake experiments, hBECs (P6) were cultured in 24-well plates (2 × 10^4^ cells/well, Corning Costar) coated with collagen type IV (100 μg/mL). The culture medium of hBECs was mixed with a PC-conditioned medium (50–50%) for 48 h. Confluent monolayers of hBECs were incubated with 3-PLG or 3-PLG-A-GSH (100 µg/mL), diluted in hBEC medium at 37 °C for 1, 4 or 24 h in a CO_2_ incubator.

To identify the mechanisms of nanocarrier cellular entry, hBECs were treated with 3-PLG or 3-PLG-A-GSH (50 µg/mL) at 4 °C for 4 h, or pre-incubated with endocytosis inhibitor randomly methylated β-cyclodextrin (5 mM, 1 h; CycloLab Cyclodextrin R&D Laboratory Ltd., Budapest, Hungary) and then incubated with nanocarriers at 37 °C for 4 h. After the incubation with nanocarriers, cells were washed three times with ice-cold phosphate buffer (PBS; KCl 2.7 mM, KH_2_PO_4_ 1.5 mM, NaCl 136 mM, Na_2_HPO_4_ × 2 H_2_O 6.5 mM, pH 7.4), supplemented with 0.1% bovine serum albumin (BSA), once with acid stripping buffer (glycine 50 mM, NaCl 100 mM, pH 3), in order to remove the cell surface-associated polypeptides, and finally once with PBS. At the end of the experiment, the hBECs were lysed in distilled water (DW) containing Triton X-100 detergent (10 mg/mL). The fluorescent signal of R6G-labeled nanocarriers was quantified from the cell lysate with a spectrofluorometer (Fluorolog 3, Horiba Jobin Yvon, Kyoto, Japan) at 525 nm excitation and with 551 nm emission wavelengths. The amount of internalized R6G-labeled nanocarriers was normalized to the protein content of cells in each well measured by BCA Protein Assay (Thermo Fischer Scientific, Waltham, MA, USA).

For the visualization of the cellular uptake of NPs, hBECs (P6) were cultured on four-chamber glass bottom petri dishes (10^5^ cells/chamber, borosilicate bottom in 35 mm dish, Greiner Bio-One, Frickenhausen, Germany) coated with Matrigel (growth factor reduced, Corning Costar). The hBECs were differentiated with 50% PC-conditioned medium (48 h before treatment), then the confluent monolayers were incubated with 3-PLG or 3-PLG-A-GSH (100 µg/mL), diluted in hBEC culture medium at 37 °C for 24 h in a CO_2_ incubator. Cell nuclei were stained with Hoechst 33342 dye (1 μg/mL, 10 min). After the incubation, the cells were washed with Ringer-HEPES buffer (118 mM of NaCl, 4.8 mM of KCl, 2.5 mM of CaCl_2_, 1.2 mM of MgSO_4_, 5.5 mM of d-glucose, 20 mM of HEPES, pH 7.4) supplemented with 1% FBS. After washing, the internalized R6G dye of the nanocarriers in the living hBECs was imaged using the 543 nm laser line on a Leica TCS SP5 confocal laser scanning microscope (Leica Microsystems, Wetzlar, Germany), equipped with an HCX PL APO 63x (NA = 1.40) oil objective. Z-stacks of 10 images, with an average thickness of 0.8 μm per slice, were generated from non-overlapping visual fields by the maximum projection of images. Mean fluorescence intensity values of images were determined using the mean gray value function in Fiji, a distribution of ImageJ2 [53]. To account for differences in background intensity and differences in cell number/image, mean fluorescence intensity values were divided by the intensity of nuclear staining or by the number of cell nuclei (Hoechst 33342, 405 nm laser line) for each image.

### 2.6. Penetration of Nanocarriers across the Co-Culture Model of Blood–Brain Barrier

For permeability studies, we prepared a human co-culture model of the BBB, in which hBECs and PCs are cultured on the opposite sides of the insert membranes [49]. First, PCs were passaged at P11 (7 × 10^3^ cells/insert) to the bottom side of the tissue culture inserts (Transwell, polycarbonate membrane, 3 μm pore size, surface 0.33 cm^2^; Corning Costar) coated with collagen type IV (100 μg/mL). Second, hBECs were seeded (2 × 10^4^ cells/insert) to the upper side of the culture insert membrane coated with Matrigel. Then, the inserts containing hBECs and PC cells on the two sides of the insert membrane were placed in 24-well plates containing endothelial culture media. The two cell types were cultured together for 7 days before permeability experiments.

The quality of the BBB model was verified by measurements of transendothelial electrical resistance (TEER) by an EVOM voltohmmeter (World Precision Instruments, Sarasota, FL, USA) combined with STX-2 electrodes. When TEER values reached a plateau level (50 ± 5 Ω × cm^2^; n = 24), indicating an appropriate tightness, the model was used for experiments. The upper donor compartment (0.2 mL) was incubated with 3-PLG or 3-PLG-A-GSH (50 µg/mL) diluted in phenol red-free DMEM/HAM’s F-12 medium (Gibco, Life Technologies, Carlsbad, CA, USA), supplemented with 1% FBS and 1% ECGS at 37 °C for 24 h on a horizontal shaker (150 rpm) in a CO_2_ incubator. To measure the integrity of the model, the transcellular marker albumin (10 mg/mL BSA + 167.5 μg/mL Evans blue; EBA, 67 kDa) and the paracellular marker sodium fluorescein (SF, 376 Da; 10 μg/mL) were also tested for permeability. After incubation, samples were collected from the donor and acceptor compartments, and the fluorescent signal of the R6G-labeled nanocarriers was quantified with a spectrofluorometer (Fluorolog 3, Horiba Jobin Yvon; excitation: 525 nm; emission: 551 nm wavelengths). The fluorescent signal of the marker molecules was quantified with the same instrument at 584 nm excitation and 663 nm emission wavelengths (EBA), and 485 nm excitation and 515 nm emission wavelengths (SF). The amount of NPs crossing the BBB model (penetration) was given as a percentage of the amount of NPs in the donor compartment (10 µg).

### 2.7. Permeability of Nanocarriers across the Blood–Brain Barrier and Internalization into Midbrain Organoids

In this experimental setup, the BBB model, hBECs (P6) and PCs (P11) cultured on inserts, as described in Section 2.6., were placed into 24-well plates. The bottom sides of the 24-well culture plates (Corning Costar, Corning, NY, USA) were replaced by borosilicate glass coverslips (VWR International, Radnor, PA, USA). Midbrain-specific organoids, derived from healthy control (control) and Parkinson’s disease patients (PD), were embedded in a Matrigel droplet (10 µL) pipetted to the glass bottom (1 organoid/well). The assembly of the BBB co-culture model with organoids is described in our previous study [52]. Donor compartments were incubated with 3-PLG or 3-PLG-A-GSH (100 µg/mL; diluted in phenol red-free DMEM/HAM’s F-12 medium, supplemented with 1% FBS and 1% ECGS, 24 h). After incubation, samples from both compartments were collected, and the fluorescent intensity of nanocarriers was measured with a spectrofluorometer (Fluorolog 3, Horiba Jobin Yvon). The apparent permeability coefficients (P_app_) were calculated as described previously [52] with the following equation:

$$\frac{\Delta {\left[C\right]}_{A}\times V_{A}}{\;A\;\times {\left[C\right]}_{D}\times \Delta t}$$

Briefly, P_app_ (cm/s) was calculated from the concentration difference in the cargo in the acceptor compartment (Δ[C]_A_) after 24 h. [C]_D_ is the concentration in the donor compartment at 0 h, V_A_ is the volume of the acceptor compartment (900 µL), and A is the surface area available for permeability (0.33 cm^2^).

To measure the penetration of nanocarriers into midbrain organoids after crossing the BBB, the organoids were homogenized in DW, containing Triton X-100 detergent (10 mg/mL), and centrifuged (13,000 rpm, 1 min, Biofuge Pico, Heraeus, Thermo Fisher Scientific, Waltham, MA, USA). After centrifugation, the fluorescent signal of the nanocarriers was quantified from the supernatant with spectrofluorometer. The protein content of the organoids was measured by using the pellets from the BCA Protein Assay kit (Thermo Fischer Scientific, Waltham, MA, USA). The nanocarrier uptake was calculated as the amount of NPs in organoids normalized to the protein content.

To visualize the entry of polypeptide NPs into midbrain organoids after crossing the BBB, the organoids were treated with Hoechst 33342 dye (2 μg/mL, 2 h) to label cell nuclei, then washed with phenol red-free DMEM/HAM’s F-12 medium (supplemented with 1% FBS and 1% ECGS). The internalized nanocarriers were visualized in living organoids by a confocal laser scanning microscope (Olympus Fluoview FV1000, Olympus Life Science Europa GmbH, Hamburg, Germany).

### 2.8. Statistics

Data are presented as means ± SD. Statistical analyses were performed using GraphPad Prism 8 software (GraphPad Software, San Diego, CA, USA). Means were compared using Student’s *t*-test, one-way or two-way ANOVA, followed by Dunnett’s post-test. Differences were considered statistically significant at *p* < 0.05. All experiments were repeated at least two times, and the number of parallel samples in each experiment was 4–24.

## 3. Results

### 3.1. Characterization of the Polypeptide Nanocarriers

**Figure 2:**
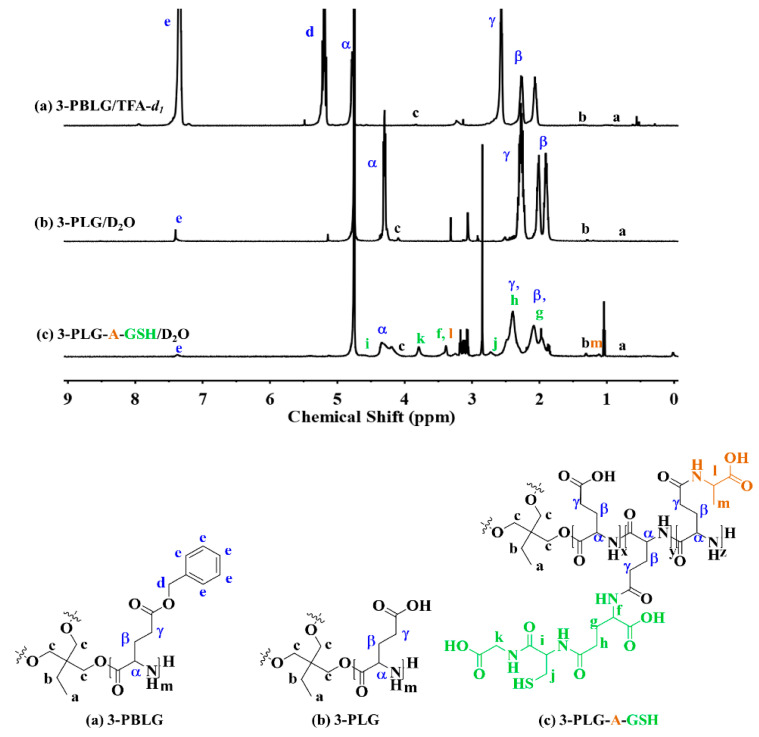
^1^H NMR spectra and structure of (**a**) 3-armed poly(γ-benzyl-l-glutamic acid (3-PBLG) in trifluoroacetic acid (TFA-*d*_1_), (**b**) 3-armed poly(l-glutamic acid) (3-PLG) in deuterium oxide (D_2_O), and (**c**) l-alanine and glutathione-targeted 3-armed poly(l-glutamic acid) (3-PLG-A-GSH) in D_2_O.

The proton nuclear magnetic resonance (^1^H NMR) analysis of 3-PBLG, 3-PLG and 3-PLG-A-GSH nanocarriers is shown in Figure 2a–c.

Based on the gel permeation chromatography–light scattering (GPC–LS) analysis, the number average molecular weight (M_n_) and polydispersity (M_w_/M_n_) of 3-PBLG were 13,600 and 1.08, respectively. The degree of polymerization was 20.5 for each arm. The success of deprotection was demonstrated by the percentage of the remaining benzyl groups on the ^1^H NMR spectrum (lower than 5%), as shown in Figure 2b. The EDC/NHS coupling reaction was used to graft A and GSH as the functional groups onto PLG segments for the synthesis of 3-PLG-A-GSH. Based on ^1^H NMR analysis, the grafting ratios of the A and GSH groups were calculated by the integrated areas of α proton (-COC*H*(CH_3_)NH-, l) on A, the methylene group (-COC*H*_2_NH-, k) on GSH and α proton (-COC*H*(R)NH-, α) on PLG (Figure 2c). The grafting ratios of A and GSH were calculated to be 10.0% and 13.9%, respectively.

The hydrodynamic diameter, polydispersity index, and zeta potential of polypeptide nanocarriers are summarized in Table 1.

The average diameter of 3-PLG was 263 nm, and in the case of targeted 3-PLG-A-GSH, it was 185 nm. The polydispersity index showed low values (0.39) in both groups, indicating a relatively narrow size distribution of nanocarriers. The zeta potential of 3-PLG was negative (−26 mV), while the surface charge of 3-PLG-A-GSH increased (−14 mV) due to the functionalization with targeting ligands.

**Figure 3:**
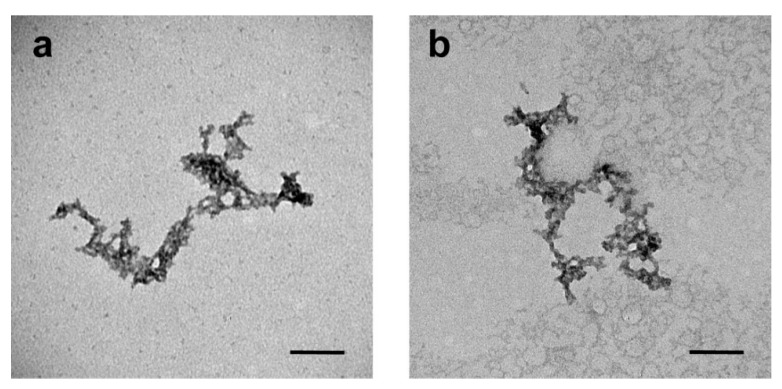
Transmission electron microscopy images of (**a**) non-targeted (3-PLG) and (**b**) alanine–glutathione-targeted (3-PLG-A-GSH) nanocarriers. Scale bar: 100 nm.

The transmission electron microscopy images show the filamentous, branched structure of the 3-PLG and 3-PLG-A-GSH samples (Figure 3a,b). Both types of nanocarriers exhibited a similar shape, but the structure of peptides is highly influenced by the sample preparation steps (treatment with ethanol, drying) for electron microscopy imaging.

### 3.2. Effect of Polypeptide Nanocarriers on the Viability of Brain Endothelial Cells

**Figure 4:**
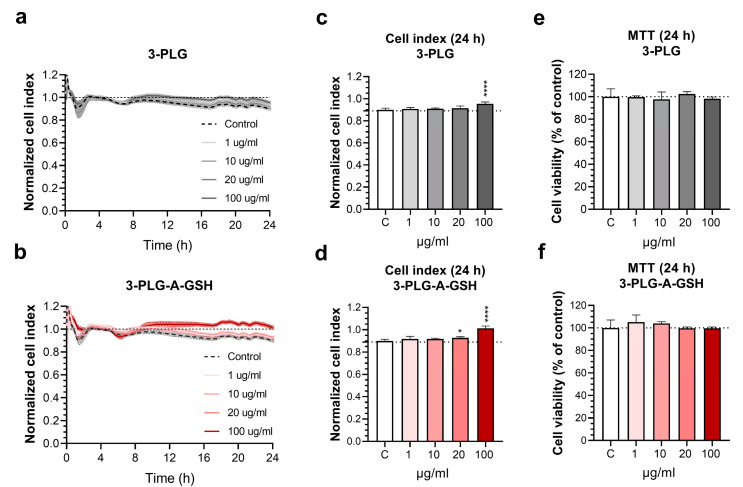
Effect of non-targeted (3-PLG) and alanine–glutathione-targeted (3-PLG-A-GSH) nanocarriers on the viability of human brain endothelial cells. Impedance kinetics of cell responses to (**a**) 3-PLG and (**b**) 3-PLG-A-GSH monitored for 24 h by real-time measurements. Impedance of human brain endothelial cells incubated with (**c**) 3-PLG and (**d**) 3-PLG-A-GSH at the 24-h time point. (**a**–**d**) Values presented are means ± SD and are given as cell index. Effect of (**e**) 3-PLG and (**f**) 3-PLG-A-GSH on the cell viability at 24 h measured by MTT test. Values presented are means ± SD and are given as a percentage of the control group. Statistical analysis: one-way ANOVA followed by Dunnett’s post-test; * *p* < 0.05, **** *p* < 0.0001, compared to the control group; n = 6–8.

The cellular effects of 3-PLG and 3-PLG-A-GSH nanocarriers on hBECs were monitored by real-time impedance measurements in the concentration range of 1–100 µg/mL for 24 h (Figure 4a,b).

In this period of time, we did not detect a decrease in the impedance of cell layers, reflecting good cell viability and maintenance of the barrier properties. The cell index was not significantly reduced in comparison to the control group receiving culture medium at 24 h. (Figure 4c,d). This was verified with the colorimetric MTT test, demonstrating that the nanocarriers had no toxic effects on the viability of hBECs after 24 h in the investigated concentration range (Figure 4e,f). We concluded that both nanocarriers can be used for further experiments at ≤100 µg/mL concentrations without cell toxicity.

### 3.3. Cellular Uptake of Polypeptide Nanocarriers, Visualization and Mechanisms of Internalization

**Figure 5:**
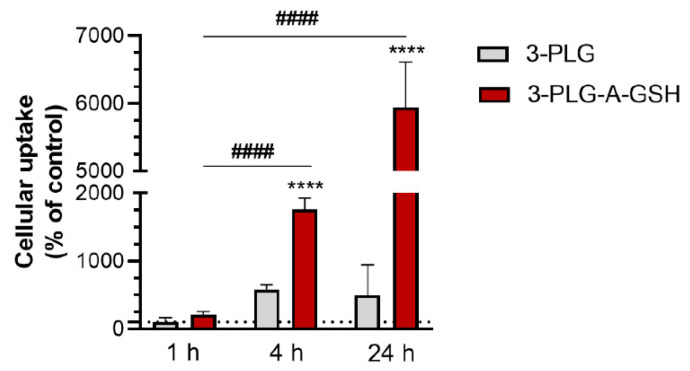
Cellular uptake of non-targeted (3-PLG) and alanine–glutathione-targeted (3-PLG-A-GSH) polypeptide nanocarriers in cultured human brain endothelial cells after 1, 4 and 24 h of incubation (100 µg/mL; 37 °C). Values presented are means ± SD and are given as a percentage of the 3-PLG group’s 1 h time point. Statistical analysis: two-way ANOVA; **** *p* < 0.0001 compared to the 3-PLG group at each time point; #### *p* < 0.0001 compared to the 3-PLG-A-GSH group at the 1 h time point; n = 6.

The time dependence of 3-PLG or 3-PLG-A-GSH internalization was monitored using the 100 µg/mL concentration at the 1, 4 and 24 h time points. The uptake of 3-PLG-A-GSH polypeptides in hBECs after 4 h showed a significant, 8 times elevation compared to the 1 h group, and its cellular entry was 3 times higher than that of the non-targeted 3-PLG group at the same time point (4 h). A further 3-fold increase in the cellular internalization of 3-PLG-A-GSH was measured at 24 h, in comparison to the 4 h time point (Figure 5).

**Figure 6:**
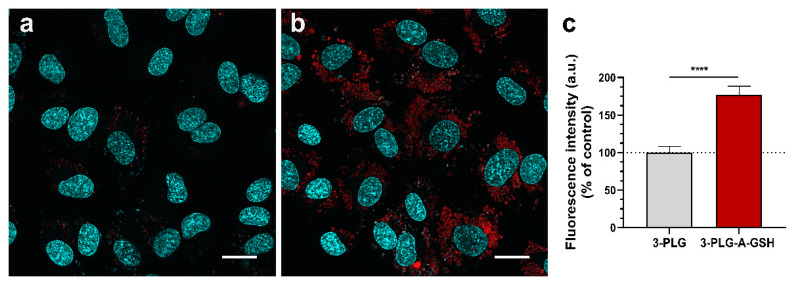
Confocal microscopy images showing the entry of (**a**) non-targeted (3-PLG) and (**b**) alanine–glutathione-targeted (3-PLG-A-GSH) nanocarriers labeled with R6G (red) into living human brain endothelial cells (100 µg/mL; 24 h incubation). Cell nuclei are stained with Hoechst 33,342 (blue). Scale bar: 20 μm. (**c**) Evaluation of the R6G fluorescence intensity of cells incubated with 3-PLG or 3-PLG-A-GSH (24 h). Values are means ± SD and given as arbitrary units (a.u.), shown as percentage of the 3-PLG group. Statistical analysis: unpaired *t*-test, **** *p* < 0.0001, compared to the 3 PLG group; n = 4.

In concordance with the results of the uptake experiment, the high-level internalization of 3-PLG-A-GSH was also verified by confocal microscopy. After 24 h incubation with 3-PLG, a low R6G signal could be detected in the cytoplasm of hBECs (Figure 6a), compared to the intensive R6G fluorescence of the dual-targeted PLG-A-GSH (Figure 6b). The results of image analysis also verify that the cellular entry of dual-targeted nanocarriers is significantly higher (1.7-times elevation) than the uptake of non-targeted 3-PLG (Figure 6c).

**Figure 7:**
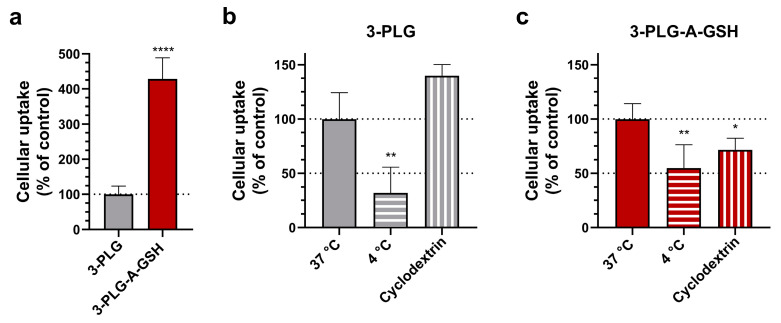
Cellular uptake and mechanism of polypeptide nanocarrier cell entry. (**a**) Uptake of 3-PLG and 3-PLG-A-GSH nanocarriers in human brain endothelial cells (4 h; 50 µg/mL). Values are means ± SD and given as a percentage of the non-targeted 3-PLG data. Statistical analysis: unpaired *t*-test, **** *p* < 0.0001, compared to the 3 PLG group. The effect of low temperature (4 °C) and endocytosis inhibitor randomly methylated β-cyclodextrin (5 mM) on the uptake of (**b**) 3-PLG and (**c**) 3-PLG-A-GSH. Values are means ± SD and given as a percentage of the control group (37 °C data, no inhibition). Statistical analysis: one-way ANOVA, Dunnett test, * *p* < 0.05, ** *p* < 0.01 compared to the respective control groups of 3-PLG and 3-PLG-A-GSH treatments; n = 6.

To reveal the mechanisms of cellular uptake, we used low temperature (4 °C) and randomly methylated β-cyclodextrin to block endocytosis (Figure 7). In these experiments, a 4 h timepoint and a lower concentration of nanocarriers (50 µg/mL) were used. Randomly methylated β-cyclodextrin (5 mM, 1 h pretreatment), which inhibits endocytosis by the selective extraction of cholesterol from the plasma membrane [54], significantly decreased the cellular internalization of 3-PLG-A-GSH compared to the group that did not receive inhibitor (Figure 7c). No effect was seen in the case of non-targeted nanocarriers (Figure 7b). At 4 °C, a low-level internalization of 3-PLG and 3-PLG-A-GSH was measured (Figure 7b,c).

### 3.4. Penetration of Nanocarriers across the Co-Culture Model of Blood–Brain Barrier

**Figure 8:**
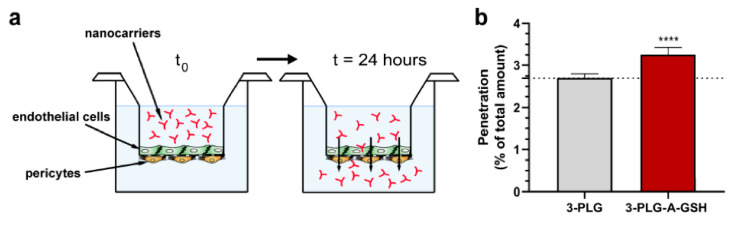
Penetration of non-targeted (3-PLG) and alanine–glutathione-targeted (3-PLG-A-GSH) nanocarriers across the human co-culture model of the blood–brain barrier (24 h incubation, 50 µg/mL, 37 °C). (**a**) Schematic drawing of the experimental set-up. (**b**) Penetration of 3-PLG and 3-PLG-A-GSH across the blood–brain barrier model. Values are means ± SD and given as a percentage of the total nanocarrier amount in the upper, donor compartment at t = 0. Statistical analysis: unpaired *t*-test; **** *p* < 0.0001; n = 6.

The penetration of polypeptide nanocarriers was investigated on a human co-culture BBB model. The schematic drawing of the model and the set-up of the experiment is presented on Figure 8a. In this assay, the transfer of nanocarriers from the upper, donor compartment to the bottom, acceptor compartment, representing the blood and brain sides, respectively, was measured. The dual-targeted 3-PLG-A-GSH crossed the BBB model in a significantly higher amount (3.25% of the total amount in the donor compartment) than the non-targeted polymer (2.71% of the total) during the 24 h assay (Figure 8b).

### 3.5. Entry of Nanocarriers into Midbrain Organoids after Crossing the Blood–Brain Barrier

**Figure 9:**
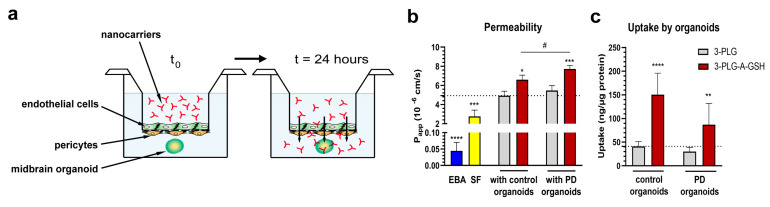
Permeability of non-targeted (3-PLG) and alanine-glutathione-targeted (3-PLG-A-GSH) nanocarriers across the human co-culture model of the blood–brain barrier and entry into midbrain-specific organoids (24 h, 100 µg/mL, 37 °C). (**a**) Schematic drawing of the experimental set-up. (**b**) Permeability of Evans blue-albumin (EBA) and sodium fluorescein (SF) reference marker molecules across the BBB model. Permeability of 3-PLG and 3-PLG-A-GSH across the co-culture model in the presence of midbrain-specific organoids derived from healthy control and from Parkinson’s disease (PD) patients’ cells. (**c**) Cellular uptake of 3-PLG and 3-PLG-A-GSH by organoids after crossing the blood–brain barrier. Values are means ± SD, n = 6. Statistical analysis: two-way ANOVA, Dunnett test. * *p* < 0.05, ** *p* < 0.01, *** *p* < 0.001, **** *p* < 0.0001 compared to the 3-PLG data in both organoid groups; # *p* < 0.05 compared between organoid groups. Permeability values of EBA and SF were compared to the 3-PLG group with control organoids (*** *p* < 0.001, **** *p* < 0.0001; one-way ANOVA, Dunnett test). P_app_: apparent permeability coefficient.

To assess the P_app_ of NPs, and to determine the uptake of nanocarriers that crossed the BBB model and subsequently entered midbrain organoids, we used an experimental set-up shown in Figure 9a. The control and PD midbrain-specific organoids were placed in the wells of the cell culture plates, below the culture inserts. To reveal the integrity of the BBB model, the permeability of the large biomarker EBA (P_app_: 0.04 × 10^−6^ cm/s) and the small paracellular marker SF (P_app_: 2.22 × 10^−6^ cm/s) was measured (Figure 9b), using the same assay conditions as for the NPs (24 h). The P_app_ values indicate that the tightness of the human BBB model was suitable for nanocarrier permeability assays.

The P_app_ values of the non-targeted polymers were in the same range and did not differ significantly between the BBB models and the control or PD organoids in the acceptor compartment (Figure 9b). We measured a significantly higher permeability for the dual-targeted nanocarriers in both groups: in the presence of control organoids, the permeability of 3-PLG-A-GSH was 6.60 × 10^−6^ cm/s (vs. 3-PLG: 4.93 × 10^−6^ cm/s), while in the presence of PD organoids, we measured 7.72 × 10^−6^ cm/s (vs. 3-PLG: 5.46 × 10^−6^ cm/s). The BBB crossing of 3-PLG-A-GSH was also significantly higher in the PD organoid group, compared to the control organoid group.

In concordance with the results of the permeability assays, the uptake of dual-targeted nanocarriers that crossed the BBB model was significantly elevated both in the control (150.49 ng/µg protein) and PD organoids (87.01 ng/µg protein), compared to the uptake of the non-targeted nanocarriers in the control (41.14 ng/µg protein) or in the PD organoids (30.31 ng/µg protein) (Figure 9c).

**Figure 10:**
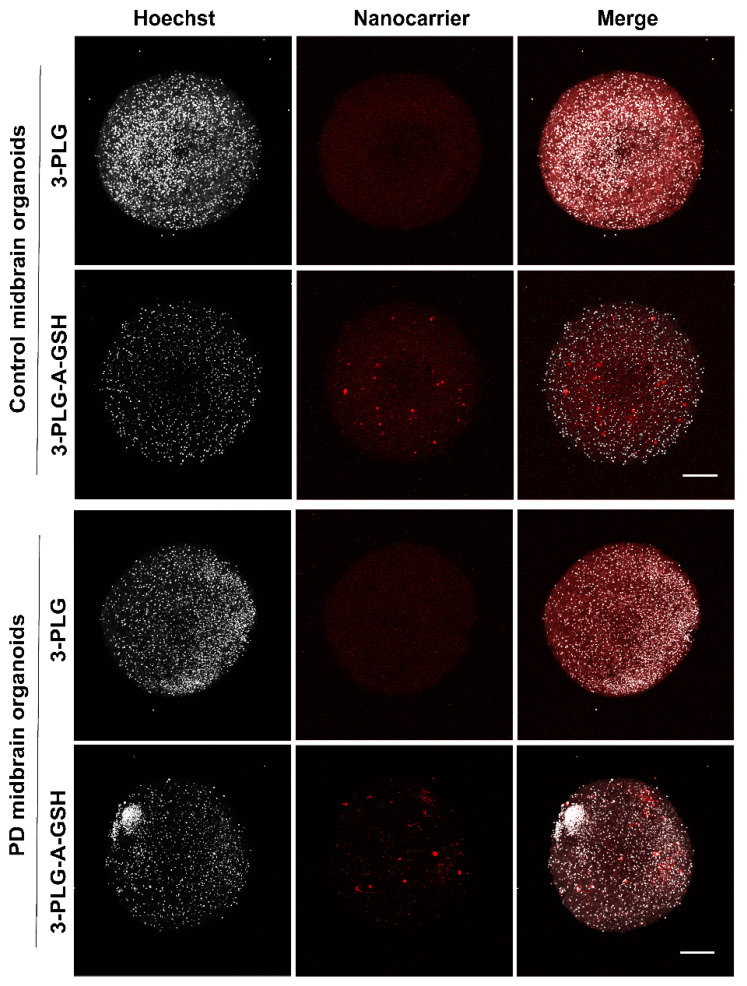
Representative confocal fluorescent microscopy images showing the uptake of non-targeted (3-PLG) and alanine–glutathione-targeted (3-PLG-A-GSH) nanocarriers (red) by midbrain-specific organoids derived from healthy (control) patients and from Parkinson’s disease (PD) patients’ cells after crossing the blood–brain barrier model (24 h, 37 °C). Cell nuclei are stained by Hoechst 33342 (blue). Scale bar: 200 μm.

The uptake of polymer nanocarriers into midbrain organoids after crossing the BBB was visualized by confocal microscopy (Figure 10). In agreement with the uptake data shown in Figure 9c, a more intensive red fluorescent signal can be seen on the representative images showing brain organoids from healthy cells or PD organoids when dual-targeted 3-PLG-A-GSH nanocarriers were used in the experiments; this is in comparison to the non-targeted 3-PLG groups (Figure 10).

## 4. Discussion

Polypeptide nanocarriers constitute a multifunctional platform to design state-of-the-art drug delivery systems. Among the polymeric NPs, poly(lactide-co-glycolide) nanocarriers coated with polysorbate 80 or poloxamer 188 have been investigated for drug delivery to the CNS [15] and have been tested on a human BBB co-culture model [55]; however, few data are available for poly(glutamic acid) NPs in this respect. PLG nanoconjugates are water-soluble, biocompatible, biodegradable and non-immunogenic nanocarriers with a high drug loading capacity and a low preparation cost. PLGs are suitable for the delivery of lipophilic drugs, such as paclitaxel [56], hydrophilic molecules, such as dopamine [12], or nucleic acids [57]. Non-targeted, star-shaped PLGs accumulate mainly in lymph nodes and the kidney, and do not reach the brain after intravenous injection in mice [58]. In contrast, PLG nanoconjugates, targeted with Angiopep-2, a ligand of the low-density lipoprotein receptor-related protein, show an increased passage across the BBB; in addition, the Angiopep-2-targeted genistein-PLG nanoconjugates accumulate in brain regions of APP/PS1 transgenic mice and exert therapeutic effects in this Alzheimer’s disease model [7]. These studies also support the importance of the presence of BBB-specific targeting molecules on PLG-based nanocarriers.

In our previous studies, we identified A and GSH as effective targeting molecules for vesicular NPs in dual combination, using rat BBB models [43,44]. In the present research, PLG nanocarriers were used, and targeting molecules A and GSH were conjugated to the polymers during the preparation. The main differences between the physico-chemical properties of the nanocarrier used in the present work and our previous studies are shown in Table 2. The PLG nanoconjugates were synthetized with the common ring-opening polymerization method and the deprotection of the benzyl groups of polypeptides [11,12]. After these steps, both the targeting molecules were efficiently conjugated to the 3-armed polymer structure based on the NMR analysis. The grafting ratios of A and GSH were ≥10%.

Both types of nanocarriers showed similar, filamentous and branched-shaped transmission electron microscopy images, but these self-assembled structures of peptide nanocarriers did not maintain their original morphology upon drying. This is mainly due to the fact that the peptide assemblies are formed via non-covalent interactions, including hydrophobic and hydrogen-bonding interactions. Since nanocarriers with an average size in the 1–300 nm range are investigated for brain targeting [1], the diameter of 3-PLG-A-GSH targeted polymers (~185 nm) makes our drug delivery system suitable for CNS applications.

The highly negative surface charge of brain endothelial cells is an important element of the defense systems of the BBB [16]. The zeta potential of NPs is a fundamental physico-chemical property that influences their interaction with biological membranes. When the surface charge of brain microvascular endothelial cells is elevated by cationic lipids or by the removal of glycocalyx residues, the cellular uptake of targeted NPs increases [43,52]. The negative zeta potential of 3-PLG (−26 mV) was increased after grafting the nanocarriers with targeting ligands (3-PLG-A-GSH: −14 mV). The slightly negative surface charge of nanocarriers can promote their BBB crossing, compared to the highly cationic or anionic NPs; however, functionalization with ligands of specific transport systems is more important for the successful brain delivery of NPs than zeta potential [16].

The cellular effects, internalization and transfer of the new polypeptide NPs was investigated on a human BBB model. The main differences between the culture BBB models used in the present work and our previous studies to investigate A–GSH-targeted NPs are shown in Table 3.

The polypeptide nanocarriers in the investigated concentration range did not show cellular toxicity, measured by impedance kinetics and MTT assay. We found a time-dependent elevated internalization of the targeted 3-PLG-A-GSH nanocarrier in hBECs, hen compared to the non-targeted 3-PLG, in agreement with our previous studies using similarly functionalized vesicular NPs [43,44]. The cellular uptake of the polypeptide nanocarriers was not only measured by the sensitive method of spectrofluorometry (Figure 5 and Figure 7), but also determined by a semi-quantitative method, namely the fluorescence intensity analysis of confocal microscope images (Figure 6). Although the direction of the changes, namely, an increase in the internalization of the targeted NPs, was similar in all the uptake experiments, the two different methods resulted in differences between the level of the endothelial uptake of the 3-PLG-A-GSH nanocarrier. The cellular uptake of NPs was partially inhibited by low temperature and by randomly methylated β-cyclodextrin, a non-selective, robust blocker of endocytosis, indicating that it is an active process. Randomly methylated β-cyclodextrin extracts cholesterol from the plasma membranes of the cells, which modifies their fluidity, and inhibits the invagination of the plasma membrane and the clathrin-coated pits; therefore, it can interfere with all known endocytic pathways, including macropinocytosis [54]. Low temperature (4 °C) inhibits the active metabolism of cells and decreases membrane fluidity, resulting in the reduced endocytosis of different molecules [59]. Low temperature and endocytosis inhibitors also decreased the uptake of GSH-targeted liposomes [60] and A–GSH-targeted vesicular NPs [43,44] in rat brain endothelial cells, indicating a similar mechanism.

We suppose that complex mechanisms take part in the internalization of targeted polypeptide NPs. It is a limitation of our study that the exact endocytic pathway(s) cannot be identified based on our experiments. Chemical inhibitors of endocytosis cannot be considered pathway specific, because most of them show off-target effects on other endocytic routes [54]. The group of inhibitors that interacts with cholesterol in the biological membranes, and affects the lipid raft and caveolae-mediated endocytosis, includes randomly methylated β-cyclodextrin (used in our present study) and filipin, used in our previous studies [43,44,52]. These molecules partially inhibited the cellular uptake of A–GSH-targeted polypeptide nanocarriers (present study) and nanovesicles [43,44,52]. Cytochalasin D is a drug blocking F-actin depolymerization and membrane ruffling, thereby inhibiting (among other pathways) macropinocytosis [54]. Cytochalasin D also partially blocked the uptake of A–GSH-targeted NPs in the cells of the BBB [43,44,52]. Based on these data, we hypothesize that macropinocytosis and caveolae-mediated endocytosis (albeit the size of caveolae may be smaller than that of our NPs) can be involved in the cell entry of the targeted polypeptides. The participation of other endocytic pathways in the uptake of targeted PLGs cannot be excluded.

The combination of two (or more) well-selected molecules targeting different BBB transporters at the same time may enhance the initial docking step to the surface of brain endothelial cells that is followed by internalization via endocytosis. Alanine is transferred to the brain by several neutral amino acid transporters present at the BBB [22], and is expressed at high levels in rat and human culture models of the BBB also [21,61]. Regarding GSH, in our recent study we demonstrated that GSH-functionalized micromanipulators bind to the surface of living rat and human brain endothelial cells; we also measured the adhesion force by an optical tweezer-based method [35]. The strong adhesion of GSH to brain endothelial cells may initiate not only the endocytic process, but also the transcytosis of targeted NPs and their cargo.

Indeed, we found that the targeted 3-PLG-A-GSH nanocarrier not only entered hBECs, but crossed the BBB model in a higher amount (Figure 8) and faster (Figure 9) than the non-targeted one. The P_app_ value of the dual-targeted polymer was 3 times higher than the permeability of the small molecular marker fluorescein across the BBB model, and 16 times higher compared to the large marker albumin. The uptake and transfer of NPs is regulated by different pathways and mechanisms [62]. In general, a large amount of NPs can be internalized by cells and only a small fraction is transferred across biological barriers. The reason for this is that NPs taken up by endocytic vesicles can end up in different subcellular structures of the endo-lysosomal system [63]. There are three major endocytic pathways for internalized NPs. They can be sorted to lysosomes via early and late endosomes, resulting in the digestion of NPs. The other two routes are retrograde trafficking to the trans-Golgi network, or recycling to the cell membrane. Only a small part of endosomes will transcytose, resulting in the case of brain endothelial cell BBB penetration [63]. In brain endothelial cultures, ~4–5% of the total amount of NPs targeted with transferrin receptor antibodies was internalized, but less than 0.02% of the targeted NPs crossed the co-culture BBB model [64]. In this study, the best targeted NP showed a 1% uptake in mouse brain capillaries, while the fraction of the injected dose in the brain parenchyma was 0.4% [64]. In line with these data, we also found a higher uptake than permeability for nanovesicles targeted with three different molecules, namely ascorbic acid, leucine, and GSH, using a co-culture BBB model [52]. Our present results are consistent with these published in vitro and in vivo data. The results of our current study obtained on a human BBB model with polypeptide NPs are also in concordance with our previous data; these previous data show that the dual A–GSH labeling of vesicular NPs elevates the BBB permeability of cargo proteins (Table 2), elevates the 67 kDa Evans blue-albumin complex [43] and elevates the 26.7 kDa mCherry [44]. The P_app_ values of the targeted 3-armed polymer with R6G cargo is several times higher than the values measured in our previous studies, with targeted NPs containing large biomolecules indicating the importance of the cargo itself in nanocarrier experiments.

The final goal of targeted drug delivery to the CNS by new NPs is to develop therapeutic platforms for the treatment of patients. Drug delivery to the CNS includes at least two steps: transfer across the BBB, and then diffusion into brain parenchyma and entry to the brain cells. We wanted to study the next step after BBB transfer, the penetration of nanocarriers into brain tissue by using a state-of-the-art model, brain organoids. Brain organoids are increasingly used in the study of the neurotoxicity of NPs [65]. In our recent study, we demonstrated that vesicular NPs targeted with a triple combination of ligands of BBB nutrient transporters, ascorbic acid/vitamin C, leucine and GSH, not only crossed a rat co-culture model of the BBB, but also entered human midbrain organoids differentiated from healthy patients and PD patients’ stem cells [52].

Induced pluripotent stem cells from patients with phenotypes and genotypes of neurodegenerative diseases can be differentiated, in vitro, into physiologically relevant disease models [51]. The PD organoids used in our study harbor a triplication in the SNCA gene and show hallmarks of the disease, namely, α-synuclein aggregation, a loss of dopaminergic neurons and impairments in astrocyte differentiation [66]. Transcriptomic data demonstrate that synaptic function is impaired in these PD-specific midbrain organoids [66]. Moreover, there are alterations in synapse number and electrophysiological activity [66]. These organoids are valuable models for PD research and drug discovery [67]. In the present study, we proved that the A–GSH dual-targeted polypeptide nanocarriers entered brain organoids after crossing the human BBB model. The uptake of targeted NPs in organoids at the 24 h time point was lower than in hBECs. This can be explained by the lower concentration of the NPs in the organoid compartment at the end of the 24 h incubation. We found previously that the glial uptake of A–GSH dual-targeted nanovesicles was modest, but the uptake of these dual-targeted NPs in neurons was >200% [44]. In that model, we also demonstrated that the cargo of the dual-targeted NPs was internalized in glial cells after crossing a triple rat co-culture BBB model (Table 3). Since brain organoids contain mostly neuronal and astroglial cells, these data are also in agreement with our measurements.

In conclusion, we could prove, using a human endothelial cell and brain pericyte co-culture model of the BBB, that A–GSH can be used as a specific targeting molecule combination, in order to increase the active cellular uptake and translocation of 3-armed polypeptide nanocarriers. Furthermore, this dual combination of molecules also elevated the penetration of the nanocarrier into human midbrain organoids. The results corroborate the notion that PLGs can be used as nanocarriers for CNS application and that the appropriate combination of brain endothelial-targeting molecules can help brain delivery.

## Figures and Tables

**Table 1:** The physico-chemical properties of nanocarriers. Values presented are means ± SD.

Nanocarrier	Size (nm)	Polydispersity Index	Zeta Potential (mV)
3−PLG	263.10 ± 37.90	0.39 ± 0.01	−25.67 ± 1.57
3−PLG−A−GSH	185.13 ± 07.59	0.39 ± 0.01	−14.00 ± 0.82

**Table 2:** Main differences between the physico-chemical properties of nanoparticles in our previous works [43,44] and the present study.

Nanocarriers	Previous Papers [43,44]	Present Manuscript
Type	niosome	polypeptide
Shape	nanovesicle, spherical	3-armed, filamentous
Composition	non-ionic surfactants cholesterol	poly(L-glutamic acid γ-benzyl ester)
Ligands	dodecanoyl alanine DSPE-PEG-glutathione	l-alanine l-glutathione
Preparation	lipid film hydratation	ring opening polymerization
Size	103 and 115 nm	185 nm
Charge	−7 and −5 mV	−14 mV
Cargo	albumin (65 kDa) mCherry (27 kDa)	rhodamine 6G (0.5 kDa)
Fluorescent marker	Evans blue, mCherry	rhodamine 6G

**Table 3:** Main differences between the cell culture models in our previous works [43,44] and the present study.

Model	Previous Papers [43,44]	Present Manuscript
Species	rat	human
Endothelial cell type	primary	stem cell-derived
Co-culture with	brain pericytes and astrocytes	brain pericytes
Cellular uptake after BBB crossing	astrocytes [44]	human brain organoids

## Data Availability

The data presented in this study are available on request from the corresponding authors.
